# Memory CD8 T Cells Generated by Cytomegalovirus Vaccine Vector Expressing NKG2D Ligand Have Effector-Like Phenotype and Distinct Functional Features

**DOI:** 10.3389/fimmu.2021.681380

**Published:** 2021-06-03

**Authors:** Marko Šustić, Maja Cokarić Brdovčak, Berislav Lisnić, Jelena Materljan, Vanda Juranić Lisnić, Carmen Rožmanić, Daniela Indenbirken, Lea Hiršl, Dirk H. Busch, Ilija Brizić, Astrid Krmpotić, Stipan Jonjić

**Affiliations:** ^1^ Department of Histology and Embryology, Faculty of Medicine, University of Rijeka, Rijeka, Croatia; ^2^ Center for Proteomics, Faculty of Medicine, University of Rijeka, Rijeka, Croatia; ^3^ Heinrich Pette Institute, Leibniz Institute for Experimental Virology, Hamburg, Germany; ^4^ Institute for Medical Microbiology, Immunology and Hygiene, Technische Universität München (TUM), Munich, Germany; ^5^ German Center for Infection Research (DZIF), Partner Site Munich, Munich, Germany

**Keywords:** memory T cells, CD8 T lymphocytes, cytomegalovirus, vaccine vector, tumor vaccine, Klrg1, TCF1

## Abstract

Viral vectors have emerged as a promising alternative to classical vaccines due to their great potential for induction of a potent cellular and humoral immunity. Cytomegalovirus (CMV) is an attractive vaccine vector due to its large genome with many non-essential immunoregulatory genes that can be easily manipulated to modify the immune response. CMV generates a strong antigen-specific CD8 T cell response with a gradual accumulation of these cells in the process called memory inflation. In our previous work, we have constructed a mouse CMV vector expressing NKG2D ligand RAE-1γ in place of its viral inhibitor m152 (RAE-1γMCMV), which proved to be highly attenuated *in vivo*. Despite attenuation, RAE-1γMCMV induced a substantially stronger CD8 T cell response to vectored antigen than the control vector and provided superior protection against bacterial and tumor challenge. In the present study, we confirmed the enhanced protective capacity of RAE-1γMCMV as a tumor vaccine vector and determined the phenotypical and functional characteristics of memory CD8 T cells induced by the RAE-1γ expressing MCMV. RNAseq data revealed higher transcription of numerous genes associated with effector-like CD8 T cell phenotype in RAE-1γMCMV immunized mice. CD8 T cells primed with RAE-1γMCMV were enriched in TCF1 negative population, with higher expression of KLRG1 and lower expression of CD127, CD27, and Eomes. These phenotypical differences were associated with distinct functional features as cells primed with RAE-1γMCMV showed inferior cytokine-producing abilities but comparable cytotoxic potential. After adoptive transfer into naive hosts, OT-1 cells induced with both RAE-1γMCMV and the control vector were equally efficient in rejecting established tumors, suggesting the context of latent infection and cell numbers as important determinants of enhanced anti-tumor response following RAE-1γMCMV vaccination. Overall, our results shed new light on the phenotypical and functional distinctness of memory CD8 T cells induced with CMV vector expressing cellular ligand for the NKG2D receptor.

## Introduction

In recent decades, various attempts have been made to harness the body’s immune system in the fight against neoplastic cells by modulating various stages of the “cancer-immunity cycle” (1). Live replicating viral vectors, genetically engineered to express tumor epitopes, have a great potential in inducing potent and long-lasting cellular immunity against malignant cells. In this respect, cytomegalovirus (CMV) represents a particularly attractive viral vector candidate due to its life-long persistence and strong capacity to induce antigen-specific CD8 T cells, which gradually accumulate in the host (2). Moreover, CMV possesses many immunomodulatory genes that can be manipulated to modulate the immune response against viral or vectored epitopes (3).

RAE-1γ is a ligand of NKG2D, an activating receptor expressed on various immune cells, including NK and CD8 T cells (4). In our previous research, we have constructed a mouse CMV vector expressing RAE-1γ in place of its viral inhibitor m152 (RAE-1γMCMV). This vector proved to be highly attenuated *in vivo* in both BALB/c and C57BL/6J mice (5). Furthermore, co-expression of foreign CD8 T-cell epitope with RAE-1γ in CMV vector induced an augmented CD8 T cell response (6). When tested in the murine melanoma model, the MCMV vector expressing RAE-1γ and tumor antigen proved to be superior in delaying melanoma growth compared to the control vector (7). However, the mechanisms conferring this increased protection remained unclear.

The memory population of CD8 T cells consists of three major subsets: central memory (Tcm), effector memory cells (Tem), and tissue-resident memory cells (Trm). Tcm express CD62L and CCR7, transcription factors Eomes and TCF1 and are thought to have enhanced proliferative capabilities with low cytotoxic potential. On the other hand, Tem lack CD62L and CCR7 expression, express T-bet and Blimp 1 transcription factors, and are associated with lower proliferative capabilities but are considered to be highly cytotoxic (8–10). Further work identified subpopulation in the Tem compartment of effector-like cells expressing KLRG1 and conferring greater protective capabilities in certain models of infection (11, 12). However, this division based on a handful of molecules has come under intense scrutiny, as the advances of single-cell sequencing technologies and mass cytometry showed memory CD8 T cells to be more heterogeneous than previously thought (13).

This study confirmed the superiority of MCMV vector expressing RAE-1γ in conferring protection against subcutaneous tumor challenge in both prophylactic and therapeutic settings. RAE-1γMCMV induced substantially higher numbers of epitope-specific memory CD8 T cells, which had a highly differentiated, effector-like transcriptional profile. The majority of these cells lacked the expression of the TCF1 transcription factor, produced lower amounts of cytokines but exhibited similar cytotoxic capabilities compared to OT-1 cells primed with the control vector. Overall, our study revealed that the insertion of RAE-1γ into the CMV vector leads to gross differences in transcriptomic, phenotypical, and functional profiles of memory CD8 T cells.

## Methods

### Mice, Viruses, Tumor Cell Lines and *In Vivo* Depletion

C57BL/6J, OT-1 (3831), *CD4^cre^* and *Klrk1^fl/fl^* mice were housed and bred under specific pathogen-free conditions at the Central Animal Facility, Faculty of Medicine, University of Rijeka, under the guidelines contained in the International Guiding Principles for Biomedical Research Involving Animals. *CD4^cre^* mice were kindly provided by D. Littman. *Klrk1^fl/fl^* mice were generated as described previously (14). Adult female mice (6-12 weeks old) were strictly age-matched for use in experiments. The Ethics Committee at the Faculty of Medicine, Rijeka and Ethics Committee of the Veterinary Department of the Ministry of Agriculture, Croatia approved all experiments.

MCMV-SIINFEKL and RAE-1γMCMV-SIINFEKL were constructed as described previously (6, 15). Virus stocks were prepared as previously described (16). Mice were immunized with 2 × 10^5^ PFU in the final volume of 50 μL DMEM *via* footpad (f.p.) route of injection.

E.G7-OVA cell line was kindly provided by V. Sexl (Vetmeduni, Vienna). Cells were cultured in RPMI 1640 (10% FCS) supplemented with G418 (Geneticin, Invivogen). B16-OVA cell line was kindly provided by T. Sparwasser (TWINCORE, Hannover) and cells were cultured in DMEM (10% FCS) also supplemented with G418. 10^6^ E.G7-OVA and 10^5^ B16-OVA cells were inoculated subcutaneously into an animal’s right flank in 100 µL PBS. Tumor growth was measured using digital caliper two-three times a week, and mice were sacrificed when tumors reached approximately 1000^3^ mm for ethical reasons.

In vivo CD8 T cell depletion was performed by i.p. injection of 150 μg of anti-CD8 antibody (YTS 169.4). Antibodies were administered once a week, over a period of 8 weeks.

### Flow Cytometry

Flow cytometry was performed according to the *Guidelines for the use of flow cytometry and cell sorting in immunological studies* (17). Splenocytes were isolated using a standard protocol. Briefly, mice were sacrificed, spleens harvested, and homogenized, followed by erythrocyte lysis. Blood samples were collected from a saphenous vein, followed by erythrocyte lysis. After leukocyte isolation, Fc receptors were blocked using a 2.4G2 antibody. For surface staining following antibodies were used: CD8α PerCP-Cy5.5 or SB780 (clone: 53-6.7; 1:400 or 1:200), CD45.1 e450 (clone: A20; 1:400), KLRG1 PE-e610 (clone:2F1, 1:100), CD127 APC (clone: SB/199, 1:100), CD44 A700 (clone: IM7, 1:100), PD1 PerCP-e710 (clone: RMP1-30; 1:200), TIM3 Pe-Cy7 (clone: RMT3-23; 1:100), CD27 PE-Cy7 (clone: LG.7F9; 1:200) and CD107a eF660 (clone: 1D4B; 1:400). Fixable Viability Dye (eBioscience) was used to exclude dead cells. For intracellular staining Intracellular fixation and permeabilization buffer set (eBioscience) was used along with following intracellular antibodies: IFNγ FITC (clone: XMG1.2; 1:100), TNFα PE-e610 (clone: MP6-XT22; 1:100), IL-2 PE-Cy7 (clone: JES6-5H4; 1:100), Granzyme B PE (clone: NGZB; 1:100). For transcription factor staining eBioscience Foxp3/Transcription Staining Buffer Set was used with: TCF1 A488 (clone:C63D9; 1:400), TOX PE (clone: TXRX10; 1:100), T-bet PerCP-Cy5.5 (clone:4B10; 1:400) and EOMES PE (clone:Dan11mag; 1:100). Antibody against TCF1 was produced by Cell Signaling Technology, and Invitrogen produced all other antibodies. Flow cytometry was performed on FACS Aria II, and data were analyzed using FlowJo v10 (Tree Star) software.

Biotinylated pMHC-I multimers were conjugated with streptavidin-PE, and splenocytes were stained as described previously (18).

### *In Vitro* Stimulation and Killer Assay

Mice harboring memory OT-1 cells, primed with MCMV-SIINFEKL or RAE-1γMCMV-SIINFEKL, were sacrificed and splenocytes isolated using a standard protocol. 2x10^6^ cells were then incubated for 6h with different concentrations of SIINFEKL peptide (JPT PeptideTechnologies GmbH) in RPMI 1640 (PAN-Biotech) supplemented with 10%FCS (PAN-Biotech), Brefeldin A (Invitrogen), Monensin (Invitrogen), and CD107 (Invitrogen) at 37°C.

For the *in vitro* killer assay, mice harboring memory OT-1 cells (CD45.1) were sacrificed and splenocytes isolated using a standard protocol. Splenocytes were pooled from 4-5 mice/group. CD8 T cells were purified by negative selection using magnetic beads (Miltenyi Biotec), and OT-1 (CD45.1) cells were stained with CD45.1 antibody and sorted using FACSAria II (BD) using high-speed sorting into RPMI supplemented with 20% FCS. Sorted OT-1 (CD45.1) cells were co-incubated with E.G7-OVA (CD45.2) cells for 4h in 2:1, 1:1, and 0.5:1 effector to target ratios at 37℃. After co-incubations, target cells were identified as CD45.1 negative, and viability was determined using Fixable Viability Dye (eBioscience). OT-1 cytotoxicity was calculated using following formula: [(% FVD^+^CD45.1^-^ cell-specific lysis − % FVD^+^CD45.1^-^ cell spontaneous lysis)/(100 − % FVD^+^CD45.1^-^ cell spontaneous lysis)] × 100 as described in (19).

### Adoptive Transfer Experiments

Naïve OT-1 cells were purified by negative selection using magnetic beads (Miltenyi Biotec). 10^4^ OT-1 (CD45.1) cells were adoptively transferred into naïve C57BL6/J (CD45.2) animals in 500 uL DMEM i.v. For adoptive transfer experiments, mice harboring memory OT-1 cells primed with viral vectors were sacrificed, splenocytes were isolated using a standard protocol, and CD8 T cells were purified using magnetic beads (Miltenyi Biotec). Following magnetic separation, OT-1 (CD45.1) cells were stained with CD45.1 (Invitrogen) antibody and sorted using FACSAria II (BD) using high-speed sorting into RPMI supplemented with 20% FCS. The purity and viability of sorted cells were checked immediately after sorting with PI staining and in all of the experiments exceeded 97%. Cells from 5-10 mice from each group were pooled, and 3x10^4^ cells were transferred in 500 uL DMEM i.v. into mice harboring tumors.

### RNAseq Sample Preparation and Sequencing

Splenocytes were isolated from mice immunized with MCMV-SIINFEKL or RAE-1γMCMV-SIINFEKL at day 36 post-immunization. CD8 T cells were purified using magnetic beads (Miltenyi Biotec). After magnetic beads separation, OT-1 (CD45.1) cells were stained with CD45.1 antibody (Invitrogen), high-speed sorted on FACSAria II (BD) directly into the RLT lysis buffer (QIAGEN), and their total RNA isolated using RNeasy Micro Kit (QIAGEN), according to manufacturers’ protocol. Agilent Bioanalyzer 2100 and Agilent RNA 6000 Nano Kit were used to estimate sample quality and determine the quantity of isolated RNA. Before library generation, RNA was subjected to DNAse I digestion (Thermo Fisher Scientific) followed by RNeasy MinElute column clean up (Qiagen). RNAseq libraries were generated using the SMART-Seq v4 Ultra Low Input RNA Kit (Clontech Laboratories) following the manufacturer’s recommendations. From cDNA, final libraries were generated utilizing the Nextera XT DNA Library Preparation Kit (Illumina). Concentrations of the final libraries were measured with a Qubit 2.0 Fluorometer (Thermo Fisher Scientific), and fragment lengths distribution was analyzed with the DNA High Sensitivity Chip on an Agilent 2100 Bioanalyzer (Agilent Technologies). All samples were normalized to 2nM and pooled at equimolar concentrations. The library pool was sequenced on the NextSeq500 (Illumina) in a single 1x84bp run, producing 22.5 M to 29.7 M reads per sample from a total of six mRNAseq libraries. Adapter sequences were hard-clipped from raw sequencing reads as part of the bcl2fastq pipeline (version 2.20.0.422). The overall quality of the trimmed sequences was assessed by FastQC v0.11.9. Where applicable, quality data from individual analyses were aggregated using MultiQC v1.9.

### RNAseq Data Processing and Analysis

RNASeq data processing and analysis were performed as described previously (20), with minor modifications specific to this study’s experimental system. Briefly, following quality control using FastQC v0.11.9, sequencing libraries were searched for potentially contaminating sequences against an in-house database of common contaminants using FastQ Screen v0.14.0 (21) and Bowtie 2 v2.3.5.1 (22). Quality-checked sequencing reads were then mapped to the mouse GRCm38.p6 (release M25) primary reference genome assembly (23) with STAR v2.7.6a (24–26), alignment files indexed using samtools v1.11 (27), and reads mapping to individual genes counted using featureCounts (28). Obtained uniquely mapped read counts were used for differential expression analysis, which was performed with the DESeq2 package (29), applying p_adj_ < 0.05 as a cutoff for statistical significance. Gene ontology overrepresentation analysis was performed using the clusterProfiler package v3.14.1 (30), and heatmaps were generated using R package pheatmap v.1.0.12 (https://CRAN.R-project.org/package=pheatmap).

### Statistical Analysis

Unpaired t-test, ANOVA (followed by LSD post test), and log-rank Mantel-Coy test were performed using Prism software (GraphPad Software Inc., La Jolla, CA). P<0.05 was considered statistically significant.

## Results

### RAE-1γMCMV Confers Robust Long-Term Protection Against Subcutaneous Tumor Challenges

**Figure 1:**
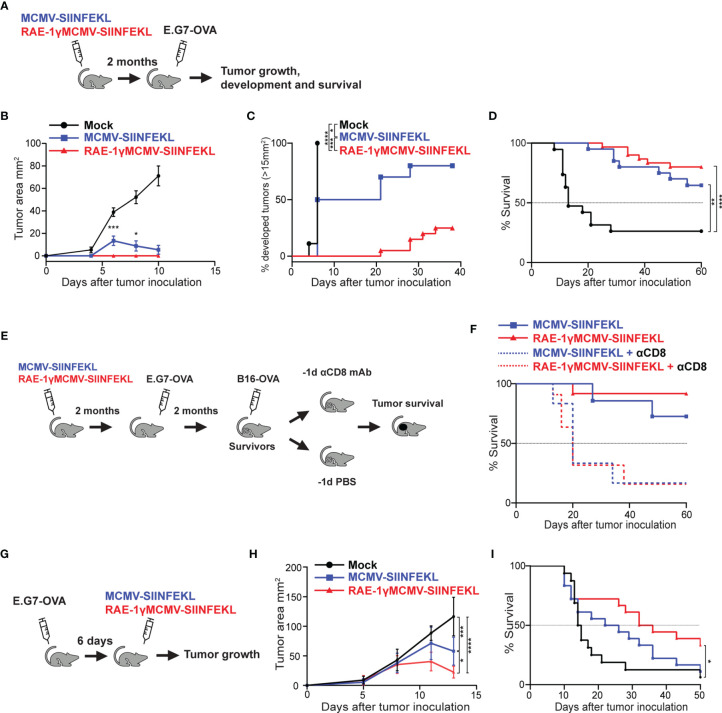
RAE-1γMCMV-SIINFEKL confers robust long-term protection against subcutaneous tumor challenges. **(A)** Mice were immunized with either MCMV-SIINFEKL, RAE-1γMCMV-SIINFEKL, or left unimmunized (n=10-20). 2 months after immunization, mice were inoculated with E.G7-OVA tumor subcutaneously. **(B)** Tumor growth was followed over time. **(C)** Percent of mice that developed tumors (>15 mm^2^). **(D)** Overall survival after E.G7-OVA challenge. **(E)** Mice that survived the initial E.G7-OVA challenge were further challenged with B16-OVA subcutaneously. Half of the survivors were depleted for CD8 T cells prior to the secondary challenge. **(F)** Overall survival of mice after secondary challenge. **(G)** Mice were inoculated with E.G7-OVA subcutaneously. 6 days later, mice were immunized with indicated vectors or left unimmunized (n=10). **(H)** Tumor growth and **(I)** overall survival were followed over time. Prophylactic data are from a single experiment representative of two independent experiments **(B, C)**. Survival data are pooled from two independent experiments **(D, F)**. Therapeutic data are from a single experiment representative of two independent experiments **(H)**, and survival data are pooled from two independent experiments **(I)**. Data are represented as mean ± SEM **(B, H)** and statistical significance is expressed as *p < 0.05, **p < 0.01, ***p < 0,001, ****p<0,0001. Statistical significance was tested using one-way ANOVA followed by LSD post test **(B, H)** or log-rank Mantel-Cox test for Kaplan-Maier curves **(C, D, F, I)**.

To expand on our previous work (6) and investigate the robustness of protection conferred to mice immunized with RAE-1γMCMV against tumor challenge, we utilized viral vectors expressing H2Kb restricted SIINFEKL epitope (6, 15). C57BL6/J mice were immunized with MCMV-SIINFEKL, RAE-1γMCMV-SIINFEKL, or left unimmunized. Two months following the immunization, mice were challenged with E.G7-OVA lymphoma expressing SIINFEKL epitope, and tumor appearance and growth were followed over time (**Figure 1A**). Unimmunized mice rapidly developed large tumors while MCMV-SIINFEKL immunized mice developed smaller tumors that subsequently retracted (**Figure 1B** **)**. Conversely, all of RAE-1γMCMV-SIINFEKL immunized mice failed to develop observable tumors during the initial three weeks after tumor inoculation, and the overall percentage of mice that developed tumors was much smaller than MCMV-SIINFEKL immunized animals (**Figure 1C** **)**. Furthermore, immunization with either of these two vectors substantially increased the overall survival of mice after the tumor challenge. Although the survival rate was higher in mice immunized with RAE-1γMCMV-SIINFEKL than in MCMV-SIINFEKL immunized mice (80% vs. 64% survival), the difference was not statistically significant (**Figure 1D** **)**. Next, two months after primary challenge, survivors of E.G7-OVA tumor were inoculated with a different, more malignant neoplasm expressing the same SIINFEKL epitope, B16-OVA melanoma (**Figure 1E**). Again, RAE-1γMCMV-SIINFEKL immunization led to an increase in the survival rate as 91% of mice survived the challenge in this group compared to 75% in the MCMV-SIINFEKL immunized group (**Figure 1F**). To confirm that the protection against secondary tumor challenge is mediated *via* CD8 T cells, half of the mice in each group were depleted of this lymphocyte population starting one day prior to the B16-OVA challenge. Mice lacking cytotoxic T cells quickly succumbed to tumor challenge, proving that this population mediates the protection.

We went on to investigate the therapeutic potential of the RAE-1γMCMV-SIINFEKL vector in the E.G7-OVA challenge. Mice were inoculated with E.G7-OVA subcutaneously and 6 days later immunized with MCMV-SIINFEKL or RAE-1γMCMV-SIINFEKL, whereas one group of mice was left unimmunized (**Figure 1G**). RAE-1γMCMV-SIINFEKL vaccination was superior to MCMV-SIINFEKL vaccination in tumor size reduction (**Figure 1H**) and substantially increased the survival of mice inoculated with E.G7-OVA lymphoma cells (**Figure 1I**). Overall, the MCMV vector expressing RAE-1γ in place of its viral inhibitor proved more efficient than the wild-type MCMV vector in protection against subcutaneous tumor challenge in both prophylactic and therapeutic settings.

### Memory CD8 T Cells Primed With RAE-1γMCMV Exhibit a Distinct Transcriptional Profile

**Figure 2:**
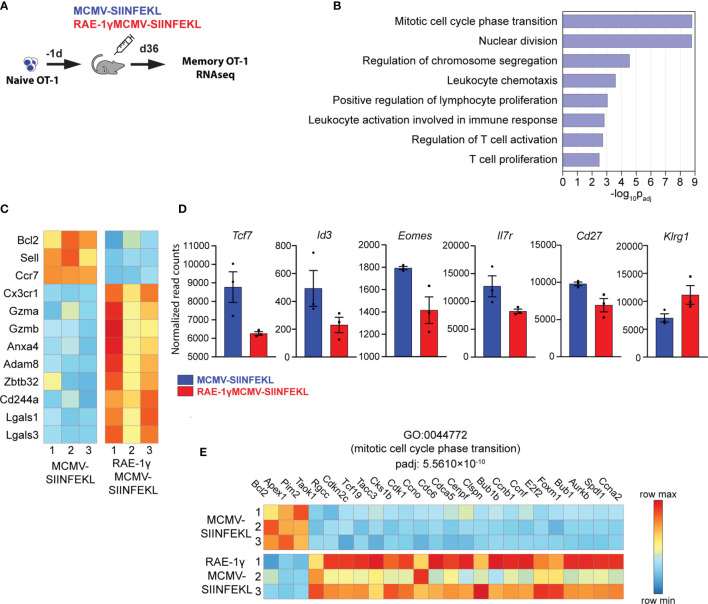
Memory CD8 T cells primed with RAE-1γMCMV exhibit a distinct transcriptional profile. **(A)** 10^4^ naive OT-1 cells were transferred to C57BL/6J mice one day prior to immunization with MCMV-SIINFEKL or RAE-1γMCMV-SIINFEKL (n=3). **(B)** Gene ontology overrepresentation analysis (GO-ORA) of identified differentially expressed genes. Bar chart showing selected, statistically significant GO terms in the Biological process category, ranked by adjusted p-values. **(C)** Heat map showing the expression of indicated genes. **(D)** Transcription of indicated genes below chosen significance threshold. **(E)** Heat map showing the expression of genes belonging to the GO term “Mitotic cell cycle phase transition”. Data are from a single experiment. Genes and GO terms with padj<0.05 were considered statistically significant.

We have previously shown that RAE1γMCMV-SIINFEKL has enhanced priming capacity compared to the wild-type MCMV vector (6, 7). However, differences in phenotype and function of memory populations of SIINFEKL specific CD8 T cells induced with these viral vectors remained poorly characterized. To screen for distinct characteristics in memory populations induced with indicated vectors, we compared their transcriptional profiles at the memory timepoint. 10^4^ congenic OT-1 T cells (expressing H-2Kb-SIINFEKL restricted TCR) were transferred to naïve animals that were subsequently vaccinated with MCMV-SIINFEKL or RAE-1γMCMV-SIINFEKL (**Figure 2A**). The transcriptional profile of OT-1 cells was determined on day 36 post-vaccination. Overall, 249 genes were differentially expressed between OT-1 cells primed with MCMV-SIINFEKL and RAE-1γMCMV-SIINFEKL (**Table S1**). Gene ontology overrepresentation analysis revealed that differentially expressed genes between these two groups participate in biological processes related to proliferation, cellular division, and cellular activation involved in immune response (**Figure 2B**). Interestingly, both *Sell* (CD62L) and *Ccr7*, coding for prototypical markers of central memory phenotype (31), as well as the antiapoptotic molecule *Bcl2*, were more strongly transcribed in the MCMV-SIINFEKL immunized group. Simultaneously, RAE-1γMCMV-SIINFEKL primed cells showed higher expression of transcripts associated with an effector-like phenotype, such as *Cx3Cr1* (32)*, Gzma, Gzmb, Adam8, Cd244a, Lgals3*, and *Lgals1* (33, 34) (**Figure 2C**). These differences prompted us to analyze other genes associated with central memory and effector-like phenotype below the chosen significance threshold. In the MCMV-SIINFEKL group, we observed a clear trend in the expression of other transcripts associated with Tcm such as *Tcf7* (35)*, Id3, Eomes, Il7r* (36), and *Cd27* (12) which were also elevated. At the same time, effector marker *Klrg1* (36) was more highly transcribed in RAE-1γMCMV-SIINFEKL immunized mice (**Figure 2D**). Furthermore, many genes linked with progression through the cell cycle were more highly transcribed in cells primed with RAE-1γMCMV-SIINFEKL, suggesting their higher proliferation rate (**Figure 2E**). Together, RNAseq data strongly associated OT-1 cells primed with RAE-1γMCMV-SIINFEKL and MCMV-SIINFEKL with effector-like and central memory phenotype, respectively.

### RAE-1γMCMV-SIINFEKL Has Superior Priming Capacity and Generates Phenotypically Distinct Memory CD8 T Cells

**Figure 3:**
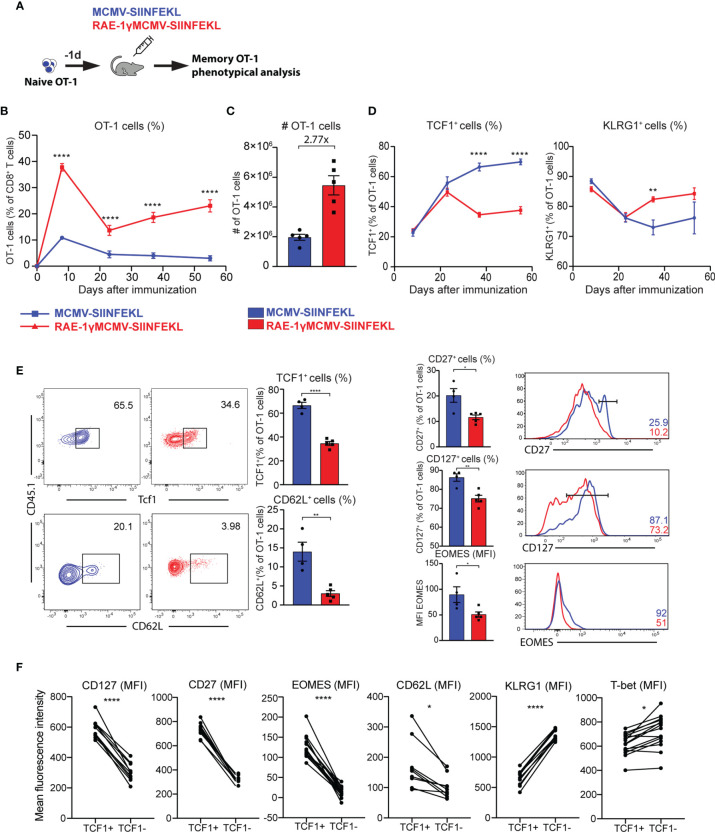
RAE-1γMCMV-SIINFEKL has superior priming capacity and generates phenotypically distinct memory CD8 T cells. **(A)** 10^4^ naive OT-1 cells were transferred to C57BL/6J mice one day prior to immunization with MCMV-SIINFEKL or RAE-1γMCMV-SIINFEKL (n=4-5). **(B)** OT-1 frequency (blood) at indicated time points post-immunization. **(C)** Absolute numbers of OT-1 cells at day 7 post-immunization in the spleen. **(D)** Kinetics of TCF1^+^ and KLRG1^+^ populations were followed in blood over time. **(E)** The phenotype of memory OT-1 cells primed with indicated viruses at day 37 post-immunization shown as the percentage of TCF1^+^, CD62L^+^, CD27^+^, CD127^+^ OT-1 cells, and MFI of Eomes on OT-1 cells. **(F)** Expression of indicated molecules on TCF1^+^ and TCF1^-^ cells. Data are from a single experiment representative of two independent experiments. Data are represented as mean ± SEM and statistical significance as *p < 0.05, **p < 0.01, ****p<0,0001. Statistical significance was determined using unpaired Student t-test.

We validated gene expression data and analyzed the kinetics and phenotype of OT-1 cells primed with viral vectors using flow cytometry. To that aim, 10^4^ congenic OT-1 T cells were transferred to naïve animals that were subsequently vaccinated with MCMV-SIINFEKL or RAE-1γMCMV-SIINFEKL (**Figure 3A**). The frequency of OT-1 T cells was followed over time. The initial expansion of OT-1 cells was substantially increased in RAE-1γMCMV-SIINFEKL immunized mice compared to MCMV-SIINFEKL immunized animals, and OT-1 T cells were maintained at significantly higher levels in the RAE1γMCMV-SIINFEKL group compared to MCMV-SIINFEKL group (**Figures 3B, C** **)**. Importantly, OT-1 cells in RAE1γMCMV-SIINFEKL showed signs of memory inflation, as the frequency of these cells increased in later time points. Confirming our RNAseq data, we observed major phenotypical differences between OT-1 cells primed with different vectors. Notably, the frequency of cells expressing the TCF1 transcription factor was significantly lower in the RAE1γMCMV-SIINFEKL immunized group than in the MCMV-SIINFEKL immunized mice (**Figure 3D**, left). RAE-1γMCMV-SIINFEKL primed OT-1 cells showed a higher frequency of KLRG1^+^ cells in accordance with RNAseq data (**Figure 3D**, right). Interestingly, these differences were not present at initial time points during acute CD8 T cell response but manifested themselves at memory time points, around day 30. A detailed phenotypical analysis of OT-1 cells at day 37 corroborated our RNAseq findings, as RAE-1γMCMV-SIINFEKL primed OT-1 cells expressed lower levels of CD62L, CD127, and CD27 as well as TCF1 and Eomes transcription factors (**Figure 3E**). To confirm that TCF1 expression successfully delineates two distinct populations with different phenotypical features, we compared the expression of several molecules on TCF1^+^ and TCF1^-^ cells. CD127, CD27, Eomes, and CD62L expression were all significantly elevated in TCF1^+^ cells, while KLRG1 and Tbet showed a significant increase in TCF1^-^ population (**Figure 3F**). These data convincingly demonstrate that RAE-1γMCMV-SIINFEKL induces memory CD8 T cells with distinct, effector-like phenotypical features that grossly differ from memory CD8 T cells primed with the vector lacking RAE-1γ expression.

### CD8 T Cells Primed With RAE-1γMCMV Show Lower Cytokine-Producing Capabilities Than Cells Primed With the Virus Lacking RAE-1γ but Similar Cytotoxic Potential

**Figure 4:**
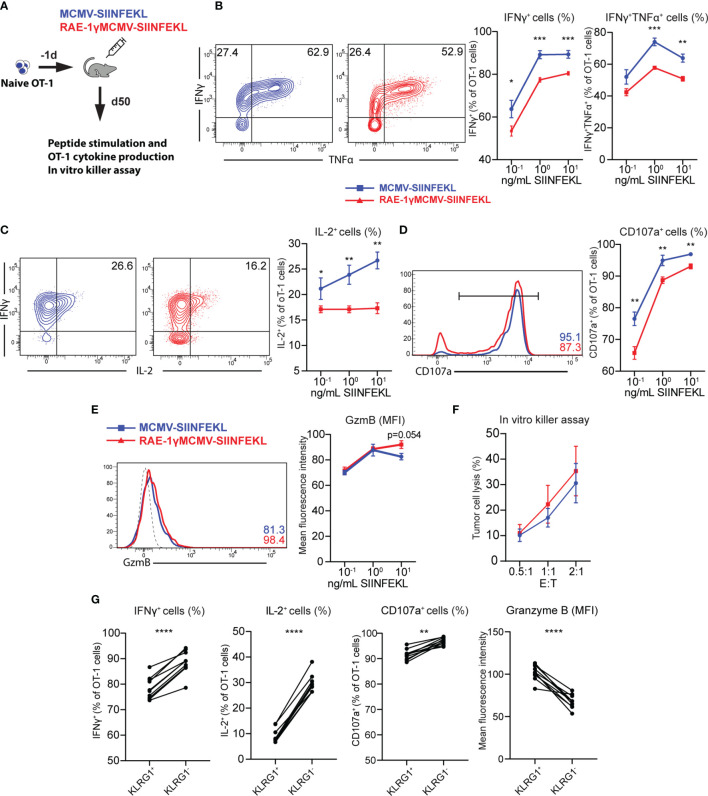
CD8 T cells primed with RAE-1γMCMV show lower cytokine-producing capabilities than cells primed with the virus lacking RAE-1γ but similar cytotoxic potential. **(A)** Naive OT-1 (CD45.1) cells were transferred to CD57BL/6J mice (CD45.2). One day after the transfer, mice were immunized with indicated viruses (n=5-6). On day 50, mice were sacrificed, and splenocytes were stimulated with different concentrations of SIINFEKL peptide. Production of IFN-γ and TNF-α **(B)**, IL-2 **(C)**, CD107a **(D)**, Granzyme B **(E)** was analyzed using flow cytometry. **(F)** Memory time point OT-1 cells were incubated with E.G7-OVA cells in indicated effector:target ratios. **(G)** Percentage or MFI of indicated molecules in KLRG1^+^ and KLRG1^-^ cells. Data are from a single experiment representative of two independent experiments. Data is represented as mean ± SEM and statistical significance *p < 0.05, **p < 0.01, ***p < 0,001, ****p<0,0001. Statistical significance was determined using unpaired Student t-test.

Distinct phenotypical features are associated with different functionality of memory CD8 T cells (31, 36). To compare the cytokine-producing capabilities of cells primed with RAE1γMCMV-SIINFEKL or MCMV-SIINFEKL on a per cell basis, OT-1 cells were transferred to naïve animals that were immunized with indicated viruses the following day. On day 50, mice were sacrificed, splenocytes were isolated and stimulated with different SIINFEKL peptide concentrations (**Figure 4A**). OT-1 cells primed with the virus lacking RAE-1γ showed higher cytokine-producing capabilities as measured by frequency of IFNγ, TNFα and IL-2 positive cells (**Figures 4B, C** **)**. Furthermore, MCMV-SIINFEKL induced memory OT-1 cells also showed enhanced degranulation ability as measured by mobilization of LAMP-1 (CD107a) molecule (**Figure 4D**). Despite the difference in CD107a mobilization, RAE-1γMCMV-SIINFEKL primed OT-1 cells showed equal or higher Granzyme B content (**Figure 4E**), suggesting the equal or higher cytotoxic potential of these cells when compared to OT-1 cells primed with wild-type virus. We sorted OT-1 cells from immunized mice at memory time point to compare the cytotoxicity of cells primed with these two vectors and incubated them in different ratios with E.G7-OVA cells. OT-1 cells primed with RAE-1γMCMV-SIINFEKL showed enhanced but not significantly different cytotoxic potential against tumor cells (**Figure 4F**), confirming previous studies that revealed that degranulation and cytotoxic potential do not necessarily correlate (37). KLRG1^+^ population was enriched in OT-1 cells primed with RAE-1γ expressing vector, and we wondered whether there was any difference in the functional capacity of this population compared to cells lacking KLRG1 expression. KLRG1^+^ cells had a lower frequency of IFNγ and IL-2 producing cells, showed lower degranulation capacity, but simultaneously had higher per cell content of cytotoxic Granzyme B molecule (**Figure 4G**). Therefore, RAE-1γMCMV-SIINFEKL primed OT-1 cells at the memory time point showed lower cytokine-producing capabilities and a lower percentage of cells mobilizing CD107a during *in vitro* stimulation. On the other hand, these cells had higher per cell Granzyme B content and similar or slightly elevated cytotoxic potential against tumor cells expressing their cognate antigen.

### Adoptively Transferred CD8 T Cells Primed With RAE-1γMCMV Vector Show Protective Potential Similar to Cells Primed With the MCMV Lacking RAE-1γ

**Figure 5:**
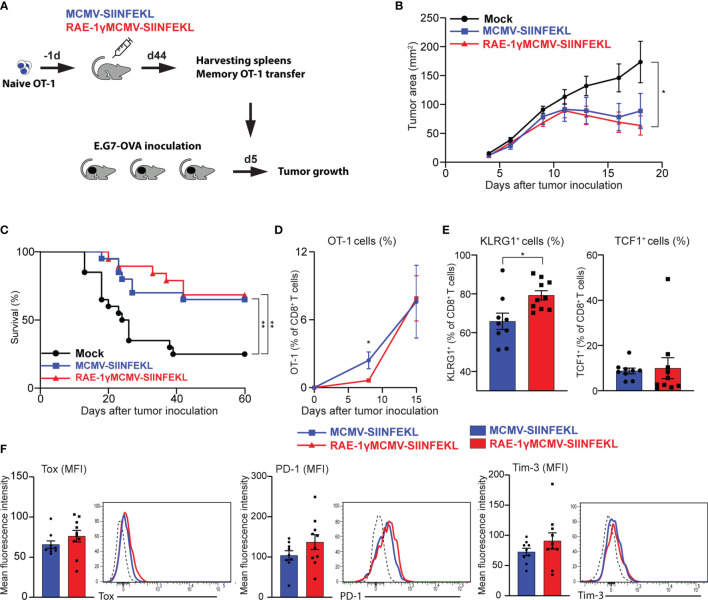
Adoptively transferred CD8 T cells primed with RAE-1γMCMV vector show protective potential similar to cells primed with the MCMV lacking RAE-1γ. **(A)** Naïve OT-1 cells were transferred into C57BL/6 mice (n=8-10) one day prior to immunization with indicated viruses. On day 44 after immunization, 3x10^4^ memory OT-1 cells were sorted and transferred to mice (n=11) that were inoculated with E.G7-OVA tumors 5 days prior to OT-1 transfer. Tumor growth **(B)** and overall survival **(C)** were followed over time. **(D)** The frequency of OT-1 cells was determined in blood at indicated time points. **(E, F)** The phenotype of OT-1 cells was analyzed on day 15 following adoptive transfer in blood. Data are from a single experiment representative of two independent experiments **(B, D–F)**. Survival data are pooled from two independent experiments **(C)**. Data are represented as mean ± SEM and statistical significance *p < 0.05; **p < 0.01. Statistical significance was determined using one-way ANOVA followed by LSD post test **(B)**, log-rank Mantel-Cox test **(C)**, or unpaired Student t-test **(D–F)**.

Prophylactic and therapeutic vaccination protocol established that immunization with RAE-1γMCMV-SIINFEKL confers greater protection against tumor challenge than immunization with a vector lacking RAE-1γ. To assess the protective capabilities of OT-1 cells generated with indicated vectors when an equal number of cells are transferred into naïve hosts, we performed an adoptive transfer experiment. OT-1 (CD45.1) cells were primed with indicated viruses, and at a memory time point, equal numbers of OT-1 cells were sorted and transferred into mice that were inoculated with E.G7-OVA tumors 5 days prior to transfer (**Figure 5A**). Both populations of transferred OT-1 cells were successful in tumor control (**Figure 5B**), and the survival rate was similar in both groups, 65% and 68% for MCMV-SIINFEKL and RAE-1γMCMV-SIINFEKL primed OT-1 cells, respectively (**Figure 5C**). Several studies associated TCF1^+^ memory cells with increased proliferative capabilities (35, 38, 39), and accordingly, the initial response was somewhat augmented in OT-1 cells primed with the wild-type MCMV vector. However, by day 15 post-transfer, the difference was lost (**Figure 5D**). In line with previous findings (40), OT-1 cells in both groups became TCF1^low^ during recall response, but RAE-1γMCMV primed cells still maintained elevated expression of KLRG1 (**Figure 5E**). We also analyzed the expression of CD8 T cell exhaustion markers PD-1 and Tim-3 on OT-1 cells, as well as Tox, a transcription factor crucially associated with T cell exhaustion (41). We found no difference in the expression of these molecules, suggesting the absence of CD8 T cell exhaustion in both groups (**Figure 5F**). Overall, OT-1 cells primed with both vectors showed comparable protective capabilities against subcutaneous tumor challenge after adoptive transfer into naive hosts.

## Discussion

In our previous work, we have demonstrated the potential of MCMV expressing NKG2D ligand RAE-1γ as a viral vaccine vector in the generation of CD8 T cell response to an inserted foreign epitope in the context of bacterial (6) and tumor challenge (7). We expanded our findings regarding the robust, long-term protection conferred by RAE-1γMCMV immunization against tumor challenge. First, we confirmed that this vector can induce substantially higher numbers of CD8 T cells specific for inserted foreign epitope than the vector lacking NKG2D ligand expression. Next, the transcriptomic analysis revealed ~250 differentially expressed genes between OT-1 cells derived from mice immunized with RAE-1γMCMV and WT MCMV vectors and uncovered that genes associated with effector-like phenotype and cellular proliferation are more strongly expressed in RAE-1γMCMV primed cells. Using flow cytometry, we validated these findings on protein levels for several genes. Remarkably, CD8 T cells induced by RAE-1γ expressing MCMV vector were predominantly TCF1 negative and showed effector-like phenotype (KLRG1^+^ CD127^-^ CD27^-^ CD62L^-^). Finally, we showed that this phenotypical difference is associated with distinct functional capabilities of cells primed with indicated vectors in a series of functional assays. RAE-1γMCMV primed OT-1 cells produced lower amounts of IFNγ, TNFα and IL-2, showed decreased degranulation potential, while simultaneously exhibiting similar cytotoxicity against tumor cells expressing their cognate antigen. However, when we transferred equal numbers of memory OT-1 cells primed with MCMV-SIINFEKL or RAE-1γMCMV-SIINFEKL into mice harboring subcutaneous tumors, OT-1 cells induced with both vectors were comparably successful in rejecting established tumors.

Memory CD8 T cells are traditionally divided by CD62L and/or CCR7 expression on central memory (Tcm) and effector memory (Tem) populations. These phenotypical properties were thought to entail functional distinction, as original reports identified the CCR7^-^ CD62L^-^ effector memory population as superior in cytokine production, whereas the central memory population was shown to exhibit enhanced proliferative capabilities (31). Further reports quickly led to a more complicated picture of functional division. For instance, Wherry et al. showed no substantial difference in cytokine-producing abilities (except IL-2 production) or cytotoxicity on per cell basis between Tcm and Tem in LCMV infection. At the same time, several other studies in different models obtained contrasting results, as Tem cells proved more protective and demonstrated higher cytotoxic capabilities (42, 43). In a recent study (11), the Tem population was further subdivided into effector-like CD127^low^ CD62L^low^ (terminal-Tem) and CD127^high^ CD62L^low^ (Tem). The effector-like terminal-Tem population (notably also expressing high levels of KLRG1) conferred the greatest protection against *Listeria monocytogenes* infection but minimal protection against tumor challenge, indicating that protective capacity is highly dependent on the disease and therapeutic context. Our transcriptomic analysis revealed several effector genes, such as *Cx3Cr1, Gzma, Gzmb, Adam8, Cd244a*, and *Lgals3*, were upregulated in CD8 T cells primed with RAE-1γMCMV, suggesting their greater cytotoxic capabilities. *Cx3cr1* is a particularly interesting gene, as one study proposed memory CD8 T cell subset classification based on the expression of this marker (32). In that study, CX3CR1^high^ cells were CD27^-^, CD127^-^ and KLRG1^+^ and produced smaller amounts of cytokines but displayed greater cytotoxic potential, therefore closely corresponding to OT-1 cells primed with RAE-1γMCMV. Interestingly, despite somewhat enhanced cytotoxicity, we observed decreased degranulation of RAE-1γMCMV primed OT-1 cells as measured by LAMP1 (CD107a) mobilization. Previous reports also demonstrated decoupling of degranulation and cytotoxicity in memory CD8 T cells specific for viral antigens, confirming that our observation is not an isolated finding (37). Furthermore, both RNAseq and flow cytometry data demonstrated elevated levels of Granzyme B in RAE-1γMCMV primed OT-1 cells indicating granule content as a better predictor of cytotoxicity than degranulation per se. This is also illustrated by the fact that KLRG1^+^ cells consistently showed lower levels of LAMP1 mobilization and substantially higher per cell content of Granzyme B.

Two aspects of CD8 T cell response are crucial for cellular immunity induced by a vaccine to be successful: cell numbers and their functionality. RAE-1γMCMV-SIINFEKL induced much higher numbers of OT-1 cells by day 7, which remained elevated throughout the experiment and showed distinct phenotypical and functional features. MCMV-SIINFEKL primed OT-1 cells, enriched in the TCF1^+^ population, appeared to have slightly augmented recall response and cytokine-producing capabilities. However, when we transferred equal numbers of OT-1 cells primed with RAE-1γ expressing vector or wild-type viral vector, both of these populations successfully rejected established tumors. Therefore, superior cytokine production and recall response of MCMV-SIINFEKL induced OT-1 cells was, perhaps, compensated by slightly elevated cytotoxicity or different tumor-infiltrating potential of RAE-1γMCMV primed OT-1 cells, leading to a similar anti-tumor response *in vivo* by adoptively transferred cells. These results indicate that the enhanced anti-tumor potential demonstrated in **Figure 1**. might depend on the immunological milieu provided by chronic/latent infection by MCMV vector expressing RAE-1γ and the superior numbers of SIINFEKL specific CD8 T cells.

The mechanistic explanation for superior priming and maintenance of CD8 T cells in RAE-1γMCMV immunized mice remains unanswered. Increased frequency of CD8 T cells might be due to the costimulatory nature of NKG2D signaling on these cells. However, several studies have shown no substantial alterations in frequency or absolute numbers of CD8 T cells that lacked NKG2D signaling during priming (44–46), and we observed no diminishment in the frequency of CD8 T cells in RAE-1γMCMV immunized NKG2D deficient mice (6), nor in conditional knock-out mice in which NKG2D receptor is specifically lacking in T lymphocytes (**Figure S1**). It is also possible that RAE-1γ mediates its function through a yet unknown interaction partner, which would explain the persistence of this phenotype even in *Klrk1^-/-^* animals. On the other hand, in RAE-1γ expressing vector, the gene is inserted in place of its viral inhibitor *m152*, which has several functions, including the retention of MHC-I molecules in ERGIC-cis Golgi compartment (47). This suggests that CD8 T cells primed with RAE-1γMCMV have stronger TCR signalling, which is known to directly correlate with the magnitude of T cell response (48, 49) and thus provides a feasible hypothesis for superior initial expansion of epitope specific CD8 T cells in RAE-1γMCMV immunized mice. However, this would only apply to CD8 T cells primed *via* direct presentation by infected dendritic cells and not during cross-presentation which was shown to have a far more important role in T cell response to MCMV epitopes (50, 51). Finally, m152 protein delays STING protein trafficking to Golgy compartment and, hence abrogates type I IFN response (52). Type I interferons are potent modulators of T cell proliferation and differentiation (53) and this increased interferon signalling could have an impact on T cell expansion in mice immunized with RAE-1γ expressing vector. Our preliminary results suggest that this is not the case, as we failed to observe any difference in T cell response to RAE-1γMCMV-SIINFEKL in STING deficient animals compared to control animals (data not shown).

Tcm phenotype is crucially connected with TCF1 expression (35, 38). Major phenotypical differences between OT-1 cells primed with RAE-1γMCMV and wild type vector regarding the expression of this transcription factor became apparent only at memory time points, around day 30. Therefore, either TCF1^+^ cells in the RAE-1γMCMV group died off or were converted to TCF1^–^ population. This conversion might be the result of an antigen encounter. Welten et al. (54) showed that TCF1^+^ cells give rise to TCF1^-^ only in the presence of latent antigenic load. Although RAE-1γMCMV establishes a lower level of latent viral load (5), immunization with this vector might lead to more frequent reactivation events or higher antigen expression on latently infected cells and higher T cell stimulation. This would not only explain the difference in phenotype between T cells primed with indicated vectors, but also signs of memory inflation in RAE-1γMCMV immunized mice and the fact that some of the most abundantly transcribed genes in this group were associated with cellular proliferation and TCR stimulation (*Lgals3* and *Zbtb32*). Another explanation for the phenotypical and functional differences might be due to different priming conditions imprinting the long-term fate of these cells on the epigenetic level. Pace et al. identified histone methyltransferases Suv39h1 as a potent epigenetic silencer of genes related to stemness/memory potential such as *Il7r, Eomes, and Ccr7* (33). Epigenetic mechanisms play a crucial role in poising the cells towards particular phenotype and functionality (55), and it would be interesting to investigate whether RAE-1γMCMV confers distinct epigenetic states to cells primed with this vector.

Overall, our study confirms great potential of highly attenuated CMV viral vectors expressing NKG2D ligand in prophylactic and therapeutic vaccine settings. The translational possibilities of CMV vectors expressing ligands for activating immune receptors are further supported by potent cellular immune response induced by human CMV vector expressing NKG2D ligand ULBP2 (56). More fundamentally, our study also indicates that small genetical changes of viral vectors can lead to gross differences in CD8 T cell expansion, phenotype, and function.

## Data Availability Statement

The RNAseq data has been uploaded to the European nucleotide archive, with accession number: PRJEB44407.

## Ethics Statement

The animal study was reviewed and approved by Ethics Committee of the Veterinary Department of the Ministry of Agriculture.

## Author Contributions

MŠ, IB, AK, and SJ designed the study. MŠ, MC, JM, CR, VL, and LH performed the experiments. MŠ and BL analyzed the data. DI performed RNA sequencing. DB provided reagents. MŠ wrote the manuscript. SJ, AK, IB, and BL critically read and revised the manuscript. SJ and AK supervised the study. All authors contributed to the article and approved the submitted version.

## Funding

This work has been supported by the grant “Strengthening the capacity of CerVirVac for research in virus immunology and vaccinology”, KK.01.1.1.01.0006, awarded to the Scientific Centre of Excellence for Virus Immunology and Vaccines and co-financed by the European Regional Development Fund (SJ), by the Croatian-Swiss Research Program of the Croatian Science Foundation and the Swiss National Science Foundation with funds obtained from the Swiss-Croatian Cooperation Programme (AK) and by the Croatian Science Foundation under the project IP-2018-01-9086 (AK).

## Conflict of Interest

The authors declare that the research was conducted in the absence of any commercial or financial relationships that could be construed as a potential conflict of interest.

## References

[1] Chen DS Mellman I. Oncology Meets Immunology: The Cancer-Immunity Cycle. Immunity (2013) 39(1):1–10. 10.1016/j.immuni.2013.07.012 23890059

[2] Klenerman P Oxenius A. T Cell Responses to Cytomegalovirus. Nat Rev Immunol (2016) 16(6):367–77. 10.1038/nri.2016.38 27108521

[3] Berry R Watson GM Jonjic S Degli-Esposti MA Rossjohn J. Modulation of Innate and Adaptive Immunity by Cytomegaloviruses. Nat Rev Immunol (2020) Feb 20(2):113–27. 10.1038/s41577-019-0225-5 31666730

[4] Raulet DH Gasser S Gowen BG Deng W Jung H. Regulation of Ligands for the NKG2D Activating Receptor. Annu Rev Immunol (2013) 31:413–41. 10.1146/annurev-immunol-032712-095951 PMC4244079 23298206

[5] Slavuljica I Busche A Babić M Mitrović M Gašparović I Cekinović Đ. Recombinant Mouse Cytomegalovirus Expressing a Ligand for the NKG2D Receptor is Attenuated and has Improved Vaccine Properties. J Clin Invest (2010) 120(12):4532–45. 10.1172/JCI43961 PMC2993599 21099111

[6] Trsan T Busche A Abram M Wensveen FM Lemmermann NA Arapovic M. Superior Induction and Maintenance of Protective CD8 T Cells in Mice Infected With Mouse Cytomegalovirus Vector Expressing RAE-1. Proc Natl Acad Sci (2013) 110(41):16550–5. 10.1073/pnas.1310215110 PMC3799388 24052528

[7] Tršan T Vuković K Filipović P Brizić AL Lemmermann NAW Schober K. Cytomegalovirus Vector Expressing RAE-1γ Induces Enhanced Anti-Tumor Capacity of Murine CD8+ T Cells. Eur J Immunol (2017) 47(8):1354–67. 10.1002/eji.201746964 PMC5672794 28612942

[8] Herndler-Brandstetter D Ishigame H Shinnakasu R Plajer V Stecher C Zhao J. KLRG1+ Effector CD8+ T Cells Lose KLRG1, Differentiate Into All Memory T Cell Lineages, and Convey Enhanced Protective Immunity. Immunity (2018) 48(4):716–29.e8. 10.1016/j.immuni.2018.03.015 29625895 PMC6465538

[9] Kaech SM Cui W. Transcriptional Control of Effector and Memory CD8+ T Cell Differentiation. Nat Rev Immunol (2012) 12(11):749–61. 10.1038/nri3307 PMC4137483 23080391

[10] Graef P Buchholz VR Stemberger C Flossdorf M Henkel L Schiemann M. Serial Transfer of Single-Cell-Derived Immunocompetence Reveals Stemness of CD8+ Central Memory T Cells. Immunity (2014) 41(1):116–26. 10.1016/j.immuni.2014.05.018 25035956

[11] Milner JJ Nguyen H Omilusik K Reina-Campos M Tsai M Toma C. Delineation of a Molecularly Distinct Terminally Differentiated Memory CD8 T Cell Population. Proc Natl Acad Sci USA (2020) 117(41):25667–78. 10.1073/pnas.2008571117 PMC7568335 32978300

[12] Olson JA McDonald-Hyman C Jameson SC Hamilton SE. Effector-Like CD8^+^ T Cells in the Memory Population Mediate Potent Protective Immunity. Immunity (2013) 38(6):1250–60. 10.1016/j.immuni.2013.05.009 PMC3703254 23746652

[13] Jameson SC Masopust D. Understanding Subset Diversity in T Cell Memory. Immunity (2018) 48(2):214–26. 10.1016/j.immuni.2018.02.010 PMC5863745 29466754

[14] Lenartić M Jelenčić V Zafirova B Ožanič M Marečić V Jurković S. NKG2D Promotes B1a Cell Development and Protection Against Bacterial Infection. J Immunol (2017) 198(4):1531–42. 10.4049/jimmunol.1600461 28087665

[15] Lemmermann NAW Gergely K Böhm V Deegen P Däubner T Reddehase MJ. Immune Evasion Proteins of Murine Cytomegalovirus Preferentially Affect Cell Surface Display of Recently Generated Peptide Presentation Complexes. J Virol (2010) 84(3):1221–36. 10.1128/JVI.02087-09 PMC2812335 19906905

[16] Brizić I Lisnić B Brune W Hengel H Jonjić S. Cytomegalovirus Infection: Mouse Model. Curr Protoc Immunol (2018) 122(1):e51. 10.1002/cpim.51 30044539 PMC6347558

[17] Cossarizza A Chang H-D Radbruch A Acs A Adam D Adam-Klages S. Guidelines for the Use of Flow Cytometry and Cell Sorting in Immunological Studies (Second Edition). Eur J Immunol (2019) 49(10):1457–973. 10.1002/eji.201970107 PMC7350392 31633216

[18] Schober K Voit F Grassmann S Müller TR Eggert J Jarosch S. Reverse TCR Repertoire Evolution Toward Dominant Low-Affinity Clones During Chronic CMV Infection. Nat Immunol (2020) 21(4):434–41. 10.1038/s41590-020-0628-2 32205883

[19] Babić M Pyzik M Zafirova B Mitrović M Butorac V Lanier LL. Cytomegalovirus Immunoevasin Reveals the Physiological Role of “Missing Self” Recognition in Natural Killer Cell Dependent Virus Control In Vivo. J Exp Med (2010) 207(12):2663–73. 10.1084/jem.20100921 PMC2989764 21078887

[20] Kveštak D Juranić Lisnić V Lisnić B Tomac J Golemac M Brizić I. NK/ILC1 Cells Mediate Neuroinflammation and Brain Pathology Following Congenital CMV Infection. J Exp Med (2021) 218(5):e20201503. 10.1084/jem.20201503 33630019 PMC7918636

[21] Wingett SW Andrews S. Fastq Screen: A Tool for Multi-Genome Mapping and Quality Control [Version 1; Referees: 3 Approved, 1 Approved With Reservations]. F1000Research (2018) 7(0):1–13. 10.12688/f1000research.15931.2 PMC6124377 30254741

[22] Langmead B Salzberg SL. Fast Gapped-Read Alignment With Bowtie 2. Nat Methods (2012) 9(4):357–9. 10.1038/nmeth.1923 PMC3322381 22388286

[23] Frankish A Diekhans M Ferreira A-M Johnson R Jungreis I Loveland J. GENCODE Reference Annotation for the Human and Mouse Genomes. Nucleic Acids Res (2019) 47(D1):D766–73. 10.1093/nar/gky955 PMC6323946 30357393

[24] Dobin A Gingeras TR. Optimizing RNA-Seq Mapping With STAR. Methods Mol Biol (2016) 1415:245–62. 10.1007/978-1-4939-3572-7_13 27115637

[25] Dobin A Gingeras TR. Mapping RNA-seq Reads With STAR. Curr Protoc Bioinformatics (2015) 51:11.14.1–11.14.19. 10.1002/0471250953.bi1114s51 26334920 PMC4631051

[26] Dobin A Davis CA Schlesinger F Drenkow J Zaleski C Jha S. STAR: Ultrafast Universal RNA-seq Aligner. Bioinformatics (2013) 29(1):15–21. 10.1093/bioinformatics/bts635 23104886 PMC3530905

[27] Li H Handsaker B Wysoker A Fennell T Ruan J Homer N. The Sequence Alignment/Map Format and SAMtools. Bioinformatics (2009) 25(16):2078–9. 10.1093/bioinformatics/btp352 PMC2723002 19505943

[28] Liao Y Smyth GK Shi W. featureCounts: An Efficient General Purpose Program for Assigning Sequence Reads to Genomic Features. Bioinformatics (2014) 30(7):923–30. 10.1093/bioinformatics/btt656 24227677

[29] Love MI Huber W Anders S. Moderated Estimation of Fold Change and Dispersion for RNA-seq Data With DESeq2. Genome Biol (2014) 15(12):550. 10.1186/s13059-014-0550-8 25516281 PMC4302049

[30] Yu G Wang L-G Han Y He Q-Y. clusterProfiler: An R Package for Comparing Biological Themes Among Gene Clusters. OMICS (2012) 16(5):284–7. 10.1089/omi.2011.0118 PMC3339379 22455463

[31] Sallusto F Lenig D Förster R Lipp M Lanzavecchia A. Two Subsets of Memory T Lymphocytes With Distinct Homing Potentials and Effector Functions. Nature (1999) 401(6754):708–12. 10.1038/44385 10537110

[32] Gerlach C Moseman EA Loughhead SM Alvarez D Zwijnenburg AJ Waanders L. The Chemokine Receptor CX3CR1 Defines Three Antigen-Experienced CD8 T Cell Subsets With Distinct Roles in Immune Surveillance and Homeostasis. Immunity (2016) 45(6):1270–84. 10.1016/j.immuni.2016.10.018 PMC5177508 27939671

[33] Pace L Goudot C Zueva E Gueguen P Burgdorf N Waterfall JJ. The Epigenetic Control of Stemness in CD8(+) T Cell Fate Commitment. Science (2018) 359(6372):177–86. 10.1126/science.aah6499 29326266

[34] Sarkar S Kalia V Haining WN Konieczny BT Subramaniam S Ahmed R. Functional and Genomic Profiling of Effector CD8 T Cell Subsets With Distinct Memory Fates. J Exp Med (2008) 205(3):625–40. 10.1084/jem.20071641 PMC2275385 18316415

[35] Zhou X Yu S Zhao DM Harty JT Badovinac VP Xue HH. Differentiation and Persistence of Memory CD8+ T Cells Depend on T Cell Factor 1. Immunity (2010) 33(2):229–40. 10.1016/j.immuni.2010.08.002 PMC2928475 20727791

[36] Martin MD Badovinac VP. Defining Memory CD8 T Cell. Front Immunol (2018) 9(NOV):1–10. 10.3389/fimmu.2018.02692 30515169 PMC6255921

[37] Wolint P Betts MR Koup RA Oxenius A. Immediate Cytotoxicity But Not Degranulation Distinguishes Effector and Memory Subsets of CD8+ T Cells. J Exp Med (2004) 199(7):925–36. 10.1084/jem.20031799 PMC2211884 15051762

[38] Jeannet G Boudousquié C Gardiol N Kang J Huelsken J Held W. Essential Role of the Wnt Pathway Effector Tcf-1 for the Establishment of Functional CD8 T Cell Memory. Proc Natl Acad Sci USA (2010) 107(21):9777–82. 10.1073/pnas.0914127107 PMC2906901 20457902

[39] Pais Ferreira D Silva JG Wyss T Fuertes Marraco SA Scarpellino L Charmoy M. Central Memory CD8+ T Cells Derive From Stem-Like Tcf7hi Effector Cells in the Absence of Cytotoxic Differentiation. Immunity (2020) 53(5):985–1000.e11. 10.1016/j.immuni.2020.09.005 33128876

[40] Lin WHW Nish SA Yen B Chen YH Adams WC Kratchmarov R. CD8+ T Lymphocyte Self-Renewal During Effector Cell Determination. Cell Rep (2016) 17(7):1773–82. 10.1016/j.celrep.2016.10.032 PMC5108530 27829149

[41] Alfei F Kanev K Hofmann M Wu M Ghoneim HE Roelli P. TOX Reinforces the Phenotype and Longevity of Exhausted T Cells in Chronic Viral Infection. Nature (2019) 571(7764):265–9. 10.1038/s41586-019-1326-9 31207605

[42] Huster KM Koffler M Stemberger C Schiemann M Wagner H Busch DH. Unidirectional Development of CD8+ Central Memory T Cells Into Protective Listeria-specific Effector Memory T Cells. Eur J Immunol (2006) 36(6):1453–64. 10.1002/eji.200635874 16637009

[43] Bachmann MF Wolint P Schwarz K Jäger P Oxenius A. Functional Properties and Lineage Relationship of CD8+ T Cell Subsets Identified by Expression of IL-7 Receptor Alpha and CD62L. J Immunol (2005) 175(7):4686–96. 10.4049/jimmunol.175.7.4686 16177116

[44] Kavazović I Lenartić M Jelenčić V Jurković S Lemmermann NAW Jonjić S. NKG2D Stimulation of CD8+ T Cells During Priming Promotes Their Capacity to Produce Cytokines in Response to Viral Infection in Mice. Eur J Immunol (2017) 47(7):1123–35. 10.1002/eji.201646805 28378389

[45] Wensveen FM Lenartić M Jelenčić V Lemmermann NAW ten Brinke A Jonjić S. NKG2D Induces Mcl-1 Expression and Mediates Survival of CD8 Memory T Cell Precursors Via Phosphatidylinositol 3-Kinase. J Immunol (2013) 191(3):1307–15. 10.4049/jimmunol.1300670 23804716

[46] Zloza A Kohlhapp FJ Lyons GE Schenkel JM Moore TV Lacek AT. NKG2D Signaling on CD8 + T Cells Represses T-bet and Rescues CD4-unhelped CD8 + T Cell Memory Recall But Not Effector Responses. Nat Med (2012) 18(3):422–8. 10.1038/nm.2683 PMC3436127 22366950

[47] Ziegler H Thale R Lucin P Muranyi W Flohr T Hengel H. A Mouse Cytomegalovirus Glycoprotein Retains MHC Class I Complexes in the ERGIC/cis-Golgi Compartments. Immunity (1997) 6(1):57–66. 10.1016/S1074-7613(00)80242-3 9052837

[48] Wherry EJ Puorro KA Porgador A Eisenlohr LC. The Induction of Virus-Specific CTL as a Function of Increasing Epitope Expression: Responses Rise Steadily Until Excessively High Levels of Epitope are Attained. J Immunol (1999) 163(7):3735–45. 10490969

[49] Wherry EJ McElhaugh MJ Eisenlohr LC. Generation of CD8 + T Cell Memory in Response to Low, High, and Excessive Levels of Epitope. J Immunol (2002) 168(9):4455–61. 10.4049/jimmunol.168.9.4455 11970989

[50] Torti N Walton SM Murphy KM Oxenius A. Batf3 Transcription Factor-Dependent DC Subsets in Murine CMV Infection: Differential Impact on T-cell Priming and Memory Inflation. Eur J Immunol (2011) 41(9):2612–8. 10.1002/eji.201041075 21604258

[51] Busche A Jirmo AC Welten SPM Zischke J Noack J Constabel H. Priming of CD8 + T Cells Against Cytomegalovirus-Encoded Antigens Is Dominated by Cross-Presentation. J Immunol (2013) 190(6):2767–77. 10.4049/jimmunol.1200966 23390296

[52] Stempel M Chan B Juranić Lisnić V Krmpotić A Hartung J Paludan SR. The Herpesviral Antagonist m152 Reveals Differential Activation of STING -Dependent IRF and NF -κB Signaling and STING ‘S Dual Role During MCMV Infection. EMBO J (2019) 38(5):1–22. 10.15252/embj.2018100983 PMC6396373 30696688

[53] Crouse J Kalinke U Oxenius A. Regulation of Antiviral T Cell Responses by Type I Interferons. Nat Rev Immunol (2015) 15(4):231–42. 10.1038/nri3806 25790790

[54] Welten SPM Yermanos A Baumann NS Wagen F Oetiker N Sandu I. Tcf1+ Cells are Required to Maintain the Inflationary T Cell Pool Upon MCMV Infection. Nat Commun (2020) 11(1):1–14. 10.1038/s41467-020-16219-3 32385253 PMC7211020

[55] Henning AN Roychoudhuri R Restifo NP. Epigenetic Control of CD8+ T’cell Differentiation. Nat Rev Immunol (2018) 18(5):340–56. 10.1038/nri.2017.146 PMC6327307 29379213

[56] Tomić A Varanasi PR Golemac M Malić S Riese P Borst EM. Activation of Innate and Adaptive Immunity by a Recombinant Human Cytomegalovirus Strain Expressing an NKG2D Ligand. PloS Pathog (2016) 12(12):1–27. 10.1371/journal.ppat.1006015 PMC5131914 27907183

