# Supplementary material for: Memory CD8 T Cells Generated by Cytomegalovirus Vaccine Vector Expressing NKG2D Ligand Have Effector-Like Phenotype and Distinct Functional Features
Source: Front Immunol. 2021 Jun 3;12:681380. doi: 10.3389/fimmu.2021.681380 (PMC8218728; doi:10.3389/fimmu.2021.681380)
Supplement: Supplementary file 1 [file DataSheet_1.docx]

Supplementary Material

**Memory CD8 T cells generated by cytomegalovirus vaccine vector expressing NKG2D ligand have effector-like phenotype and distinct functional features**

**Marko Šustić^1^, Maja Cokarić Brdovčak^2^, Berislav Lisnić^2^, Jelena Materljan^1^, Vanda Juranić Lisnić^2^, Carmen Rožmanić^2^, Daniela Indenbirken^3^, Lea Hiršl^2^, Dirk H. Busch^4,5^, Ilija Brizić^2^, Astrid Krmpotić^1^ and Stipan Jonjić^1,2^**

**^1^**Department of Histology and Embryology, Faculty of Medicine, University of Rijeka, Rijeka, Croatia

**^2^**Center for Proteomics, Faculty of Medicine, University of Rijeka, Rijeka, Croatia

**^3^**Heinrich Pette Institute, Leibniz Institute for Experimental Virology, Hamburg, Germany

^4^Institute for Medical Microbiology, Immunology and Hygiene, Technische Universität München (TUM), Munich, Germany

^5^German Center for Infection Research (DZIF), Partner Site Munich, Munich, Germany

**Keywords: memory T cells, CD8 T lymphocytes, cytomegalovirus, vaccine vector, tumor vaccine, Klrg1, Tcf1**

***Correspondence:**

Stipan Jonjić, stipan.jonjic@medri.uniri.hr

********

RAE-1γMCMV-SIINFEKL

MCMV-SIINFEKL

**Supplementary Figure 1. NKG2D expression on T lymphocytes is not necessary for superior maintenance and distinct phenotypical features of RAE-1γMCMV-SIINFEKL primed CD8 T cells.** CD4^cre^NKG2D^fl/fl^ mice or NKG2D^fl/fl^ littermates were infected with MCMV-SIINFEKL or RAE-1γMCMV-SIINFEKL. 3 months after infection SIINFEKL specific CD8^+^ T cells were analyzed in the spleen of immunized animals. (A) Percentage of SIINFEKL specific CD8^+^ T cells determined by H-2Kb-SIINFEKL multimer staining. (B-D) Percentage of SIINFEKL specific CD8 T cells expressing indicated molecules. Statistical analysis was performed with ANOVA followed by Tuky post-test. Data is represented as mean ± SEM and statistical significance *p < 0.05, **p < 0.01.
